# Nuclear IRF-1 expression as a mechanism to assess “Capability” to express PD-L1 and response to PD-1 therapy in metastatic melanoma

**DOI:** 10.1186/s40425-017-0229-2

**Published:** 2017-03-21

**Authors:** James W. Smithy, Lauren M. Moore, Vasiliki Pelekanou, Jamaal Rehman, Patricia Gaule, Pok Fai Wong, Veronique M. Neumeister, Mario Sznol, Harriet M. Kluger, David L. Rimm

**Affiliations:** 1 Department of Pathology, BML116 Yale School of Medicine, 310 Cedar Street, PO Box 208023, 06520 New Haven, CT USA; 2 Section of Medical Oncology, Yale School of Medicine, New Haven, CT USA

**Keywords:** Melanoma, PD-L1, IRF-1, Biomarkers

## Abstract

**Background:**

Predictive biomarkers for antibodies against programmed death 1 (PD-1) remain a major unmet need in metastatic melanoma. Specifically, response is seen in tumors that do not express programmed death ligand 1 (PD-L1), highlighting the need for a more sensitive biomarker. We hypothesize that capacity to express PD-L1, as assessed by an assay for a PD-L1 transcription factor, interferon regulatory factor 1 (IRF-1), may better distinguish patients likely to benefit from anti-PD-1 immunotherapy.

**Methods:**

Samples from 47 melanoma patients that received nivolumab, pembrolizumab, or combination ipilimumab/nivolumab at Yale New Haven Hospital from May 2013 to March 2016 were collected. Expression of IRF-1 and PD-L1 in archival pre-treatment formalin-fixed, paraffin-embedded tumor samples were assessed by the AQUA method of quantitative immunofluorescence. Objective radiographic response (ORR) and progression-free survival (PFS) were assessed using modified RECIST v1.1 criteria.

**Results:**

Nuclear IRF-1 expression was higher in patients with partial or complete response (PR/CR) than in patients with stable or progressive disease (SD/PD) (*p =* 0.044). There was an insignificant trend toward higher PD-L1 expression in patients with PR/CR (*p =* 0.085). PFS was higher in the IRF-1-high group than the IRF-1-low group (*p =* 0.017), while PD-L1 expression had no effect on PFS (*p =* 0.83). In a subset analysis, a strong association with PFS is seen in patients treated with combination ipilimumab and nivolumab (*p =* 0.0051).

**Conclusions:**

As a measure of PD-L1 expression capability, IRF-1 expression may be a more valuable predictive biomarker for anti-PD-1 therapy than PD-L1 itself.

**Electronic supplementary material:**

The online version of this article (doi:10.1186/s40425-017-0229-2) contains supplementary material, which is available to authorized users.

## Background

Blockade of the PD-1/PD-L1 axis has revolutionized treatment of metastatic melanoma in recent years, with five-year survival rates as high as 34% for patients treated in the initial trials of nivolumab [1]. However, the majority of this benefit is concentrated in a relatively small subset of patients. In large randomized trials, objective response rates to PD-1 blocking antibodies such as nivolumab and pembrolizumab have ranged from 28 to 40% [2–5]. Despite the efficacy of these agents, this class has been infrequently associated with severe immune-related toxicity, including pneumonitis,[6] acute kidney injury,[7] and endocrinopathies.[8, 9] Furthermore, substantially increased toxicity is observed with the combination of nivolumab and ipilimumab, which is gaining traction as a standard of care in metastatic melanoma though the mature overall survival data for combination therapy are still pending [10, 11].

Given this risk-benefit profile, there is no broadly accepted diagnostic assay to identify patients that are most likely to respond to PD-1 blockade. In melanoma and other tumor types, clinical trials have focused on immunohistochemical (IHC) staining of programmed death ligand-1 (PD-L1) as a potential predictive biomarker for response.[2] PD-L1 is one of several ligands to PD-1, and can be expressed on either tumor cells or stromal cells.[12] While PD-L1 expression in pre-treatment tumors is generally associated with higher response rates [13], the limited predictive power of this biomarker has discouraged its development as a companion diagnostic in melanoma. Specifically, it is concerning that high false-negative rates for PD-L1 companion diagnostics could exclude potential responders from anti-PD-1 therapy in a population with very limited treatment options. Dako’s 28-8 PD-L1 assay has been approved as a “complementary” diagnostic in melanoma, but PD-L1 IHC testing has not yet been incorporated into routine clinical practice in melanoma.

One potential explanation for PD-L1 protein expression’s relatively poor performance as a predictive biomarker is its markedly heterogeneous staining pattern [14]. In multiple tumor types, PD-L1 is often focally expressed in close proximity to lymphocytic infiltrates near the tumor-stromal interface [15]. In melanoma, PD-L1 expression correlates with higher CD8+ infiltrates across multiple anatomic sites [16]. These observations have been further developed into a model of adaptive immune evasion, in which secretion of interferon gamma (IFNγ) by infiltrating immune cells locally activates JAK/STAT signaling in tumor cells and induces focal expression of PD-L1 [17–20]. In this context, it is possible that spatial or temporal sampling error could account for some of the yet-unexplained responses to anti-PD-1 therapy in PD-L1 negative tumors.

Thus, we hypothesized that identifying a tumor’s capability to express PD-L1 under the appropriate conditions might identify a broader range of cases that may respond to anti-PD-1 agents than assessment of PD-L1 alone. Specifically, we considered the expression of the PD-L1 transcription factor interferon regulatory factor-1 (IRF-1) as a possible marker for this capability. IRF-1 lies immediately upstream of PD-L1 in the IFNγ-driven JAK/STAT signaling cascade [17], and has been shown to play a central role in regulating cancer cell’s response to IFNγ [21]. Unlike other components of the JAK/STAT pathway, IRF-1 is generated *de novo* in response to IFNγ binding, making it uniquely amenable to IHC assays. While it is possible that IRF-1 expression correlates with that of PD-L1, detection of this transcription factor may represent a method to determine a cell state that is capable of expression of PD-L1, when facilitated by local molecular microenvironment [22].

To evaluate IRF-1 as a predictive biomarker, we sought to quantitatively compare PD-L1 expression with a comparable IHC assay for IRF-1 in predicting response to anti-PD-1 immunotherapy. We hypothesized that high IRF-1 expression may reflect a tumor’s ability to benefit from anti-PD-1 therapy independent of its PD-L1 expression status.

## Methods

### IRF-1 and PD-L1 induction in cell lines

Melanoma cell lines were grown to 80% confluency, serum-starved for 24 h and then treated with IFNγ or control media for 24 h. Cells were then fixed directly on chamber slides, lysed for Western blotting, or fixed with formalin to generate paraffin-embedded (FFPE) pellets. Cells grown on chamber slides were washed twice in 1X phosphate-buffered saline (PBS) and then fixed in 4% paraformaldehyde (PFA) with 88 mM sucrose. For FFPE cell pellets, five ten-centimeter plates grown to confluency were first rinsed with PBS, and fixed in a solution 4% PFA at 4° Celsius overnight. Cells were then resuspended and rinsed three times in PBS before being washed twice in 80% ethanol (EtOH). Cell pellets were spun at 12,000 RPM and embedded in 2.2% melted agarose in PBS. Agarose-embedded pellets were incubated in 70% EtOH overnight and then sequentially dehydrated with one-hour incubations of 90% EtOH and 100% EtOH, two one-hour xylene washes, and submerged in molten paraffin for two hours before embedding.

### Antibody validation

**Fig. 1:**
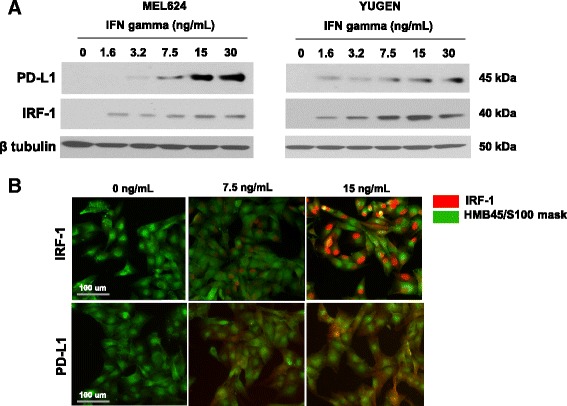
IRF-1 assay validation in cell lines and melanoma cases. **a** Induction of IRF-1 and PD-L1 with increasing concentrations of interferon gamma in YUGEN and Mel624 melanoma cell lines by Western blot. **b** Induction of IRF-1 and PD-L1 in YUGEN melanoma cells by immunofluorescence. Green (Cy3 channel) = HMB45/S100 tumor mask. Red (Cy5 channel) = target

Antibodies for IRF-1 (CST D5E4; #8478) and PD-L1 (Spring Bioscience SP142; #M4420) were validated [23] by migration on Western blot and subcellular localization with progressive expression. Upon treatment with IFNγ, melanoma cell lines upregulated IRF-1 and PD-L1 as detected by Western Blot (Fig. 1a) and immunofluorescence (Fig. 1b). Immunofluorescent staining for IRF-1 was limited to the nucleus, while PD-L1 expression was detected in the membrane and cytoplasm. Progressively increased expression of each marker seen with increased IFNγ stimulation was used to confirm specificity.

### Western blot

Cells were lysed in ice-cold M-PER mammalian Protein Extraction Reagent (Thermo Scientific) supplemented with protease inhibitors. To determine protein concentration a Bradford assay was conducted using the Bio-Rad protein assay reagent (Bio-Rad,). Proteins (30 μg) were subjected to sodium dodecyl sulfate-polyacrylamide gel electrophoresis (SDS-PAGE) and transferred to a nitrocellulose membrane (GE Healthcare). The resulting blots were blocked for 1 h at room temperature (RT) in 5% skimmed dry milk diluted in 1X Tris-buffered saline supplemented with Tween-20 (TBST). Blots were incubated at 4 °C overnight in primary antibodies specific for PDL-1 (Spring Bioscience Clone SP142; diluted 1:500) or IRF-1 (Cell Signaling Technology Clone D5E4; diluted 1:1000). Following incubation, blots were washed with 5% milk/TBST before incubation with a horseradish peroxidase, labeled goat anti-rabbit IgG (Santa Cruz Biotechnology Inc.; diluted 1:5000) at RT for 1 h. Blots were washed with 5% milk/TBST and bands were visualized using electrochemiluminescence detection reagents (Thermo Scientific).

### Case identification

Medical records and tissue samples were identified for melanoma patients with non-ocular primary tumors treated with pembrolizumab or nivolumab within the Yale-New Haven Health system before April 1, 2016 under a protocol approved by Yale Human Investigations Committee. 51 cases with available pre-treatment tissue specimens were identified and selected by a board-certified pathologist. Of these, 47 had appropriate imaging available (e.g., CT, PET, and/or MRI) to determine response and PFS by objective criteria. Of the 47 cases, 21 (45%) demonstrated a partial or complete response, including one case of pseudo-progression. Objective radiographic response (ORR) and PFS were determined by review of available CT or MRI scans using modified RECIST v1.1 criteria [24]. To account for the possibility of pseudo-progression [25], progression at first follow up scan needed to be confirmed with further progression at a second follow up scan to be classified as PD. Twenty-eight cases (60%) were treated with single-agent pembrolizumab or nivolumab and 19 cases (40%) were treated with combination ipilimumab and nivolumab. Additional cohort characteristics are described in Table 1. The frequency of responses in the monotherapy and dual therapy subgroups were 46% and 42%, and the median PFS were 5.9 and 6.1 months, respectively.

**Table 1:** Clinical and pathologic characteristics of the study cohort

		All patients	IRF-1 High	IRF-1 Low
N		47	31	16
Median age at diagnosis		62	63	60
Sex	Male	24	14	10
	Female	23	17	6
Race	White	44	30	14
	Black	2	0	2
	Hispanic	1	1	0
Treatment	Pembrolizumab	18	12	6
	Nivolumab	10	4	6
	Ipilimumab + nivolumab	19	15	4
Prior checkpoint blockade	Yes	16	11	5
	No	31	20	11
Mutation status	BRAF	16	11	5
	NRAS	6	5	1
	CKIT	2	2	0
	None detected	23	13	10
Stage at diagnosis	I	5	3	2
	II	8	5	3
	III	17	11	6
	IV	11	8	3
	Unknown	6	4	2

### Quantitative immunofluorescence

FFPE whole-tissue sections, tissue microarrays (TMAs) and cell pellets were processed and stained as previously described[26]. Briefly, sections were baked for 30 min at 60 °C and underwent two 20-min washes in xylenes. Slides were rehydrated in two 1-min washes in 100% EtOH followed by one wash in 70% EtOH and finally rinsed in streaming tap water for 5 min. Antigen retrieval was performed in sodium citrate buffer, pH 6, for 20 min at 97 °C in a PT module (LabVision). Endogenous peroxidases were blocked by 30-min incubation in 2.5% hydrogen peroxide in methanol. Subsequent steps were carried out on the LabVision 720 Autostainer (Thermo-Scientific). Nonspecific antigens were blocked by a 30-min incubation in 0.3% bovine serum albumin (BSA) in TBST. Slides were then incubated with the target primary antibody, as well as a cocktail of two mouse monoclonal antibodies against S100 (Clone 15E2E2, BioGenex) and HMB45 (Clone HMB45, Biogenex) each diluted at 1:100 to define the tumor compartment. IRF-1 was detected with rabbit monoclonal antibody clone D5E4 (Cell Signaling Technologies) at 0.6 ug/mL and PD-L1 was detected with rabbit monoclonal antibody SP-142 (Spring Biosciences) at 0.08 ug/mL.

Primary antibodies were followed by incubation with Alexa 546–conjugated goat anti-mouse secondary antibody (Life Technologies) diluted 1:100 in rabbit EnVision reagent (Dako) for 1 h. Signal was amplified with Cy5-Tyramide (Perkin Elmer) for 10 min, and then nuclei were stained with DAPI in BSA-tween for 10 min. Slides were mounted with ProlongGold (Life Technologies). Two TBS-T washes and one TBS wash were performed between each step after the primary antibody.

For cells fixed on chamber slides, samples were washed twice in PBS after fixation and permeabilized with 0.25% Triton X-100 in PBS for 10 min. Cells were washed twice in PBS and blocked with 1% BSA in PBS for 1 h at room temperature. Block was decanted off slides and the primary antibody cocktail as described above was applied. Subsequent steps were identical to the staining of FFPE tissue, except the DAPI stain was substituted for mounting Prolong Gold with DAPI (Life Technologies). One PBS-T and one PBS wash were performed between each step after the primary antibody.

Immunofluorescence was quantified using automated quantitative analysis (AQUA) on all fields of view containing tumor on each slide. Briefly, fluorescent images of DAPI, Cy3 (Alexa 546-S100/HMB45), and Cy5 (PD-L1 or IRF-1) for each field of view (FOV) were collected. Image analysis was carried out using the AQUAnalysis software (Genoptix), which generated an AQUA score for each compartment by dividing the sum of target pixel intensities by the area of the compartment in which the target is measured [27]. PD-L1 was measured in the S100/HMB45-positive tumor compartment and IRF-1 was measured within the DAPI-positive nuclear compartment within the tumor compartment. A total AQUA score was determined for each case by averaging scores from each 20X field of view.

### Chromogenic staining

FFPE cases were stained for IRF-1 as described above through the secondary antibody incubation. Then, slides were incubated with 3,3'-diaminobenzidine peroxidase substrate (Vector Laboratories) for 8 min and counterstained with Tacha’s Auto Hematoxylin (Biocare Medical). Slides were then dehydrated in washes of 70% EtOH, 100% EtOH, and xylenes before mounting. Chromogenic staining for PD-L1 was performed using the FDA-approved 22C3 assay on the DAKO Link 48 automated staining platform.

### Statistics

AQUA scores between responders (PR/CR) and non-responders (SD/PD) were compared using an unpaired *t* test; PFS and OS between groups were compared using the log-rank test. A Cox proportional hazards model was constructed with age, sex, race, mutational status, prior checkpoint blockade, and IRF-1 status. All univariate statistical analyses was performed using GraphPad Prism 7 (GraphPad Software), and multivariate analysis was performed with JMP 11 (SAS Institute). All p values reported for subset analyses are descriptive and were not adjusted for multiple comparisons. For each biomarker, the sample size of 47 patients was sufficient to detect an 83% standard-deviation difference in AQUA scores between responders (CR/PR) and non-responders (PD/SD) with 80% power at *p =* 0.05.

## Results

**Fig. 2:**
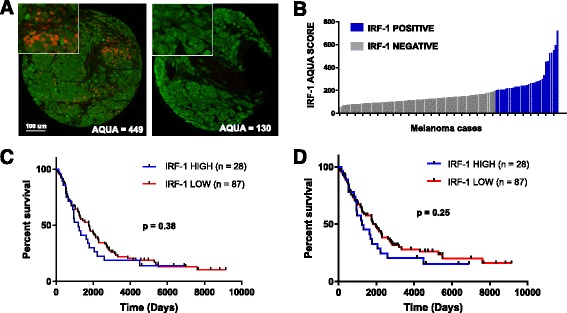
Characterization of IRF-1 in human melanoma samples. **a** Representative IRF-1-positive and IRF-1-negative melanoma cases from Yale tissue microarray (YTMA) 98. Green (Cy3 channel) = HMB45/S100 tumor mask. Red (Cy5 channel) = target. **b** Average AQUA scores for nuclear IRF-1 for 115 melanoma cases on YTMA 59. Blue bars = visible nuclear staining. Gray bars = no nuclear staining. **c** Overall survival in 115 melanoma cases unselected for treatment on YTMA 59 using visual threshold cutpoint. **d** Disease-specific survival for cases on YTMA 59 using visual cutpoint

To identify IRF-1 expression patterns in melanoma tissue, two TMAs of unselected melanoma cases (YTMA 98 and YTMA 59) were stained for IRF-1 (Fig. 2a). Of 115 tumor cases on YTMA 59, 28 exhibited identifiable nuclear staining in the tumor; average AQUA scores for these positive cases ranged from 204 to 723 (Fig. 2b). Nuclear IRF-1 staining was also observed in stromal cells in close proximity IRF-1-positive tumor cells, but this expression was not quantified in the current assay. We then sought to assess whether IRF-1 is a prognostic factor in melanoma irrespective of treatment. Cases from YTMA 59 were stratified into IRF-1-high and IRF-1-low cohorts using an AQUA cutpoint of 204 based on the threshold for visual positivity. In this cohort, IRF-1 did not predict overall survival (OS) (Fig. 2c) or disease-specific survival (Fig. 2d).

To assess IRF-1 as a predictive marker for response to PD-1 blockade, serial whole-tissue sections from 47 melanoma patients treated with anti-PD-1 immunotherapy were then stained for IRF-1 and PD-L1 in three batches. Batch-to-batch assay reproducibility was assessed by correlating scores from an index tissue microarray run with each batch (Additional file 1: Figure S1). The median number of 20X fields of view per case was 64 for IRF-1 (range: 4 - 667), and 64 for PD-L1 (range: 7 - 764). There were trends toward higher expression of both markers in metastases compared to primary tumors, and in patients treated with prior checkpoint blockade compared to patients without prior treatment, though these differences did not reach statistical significance (Additional file 1: Figure S2).

**Fig. 3:**
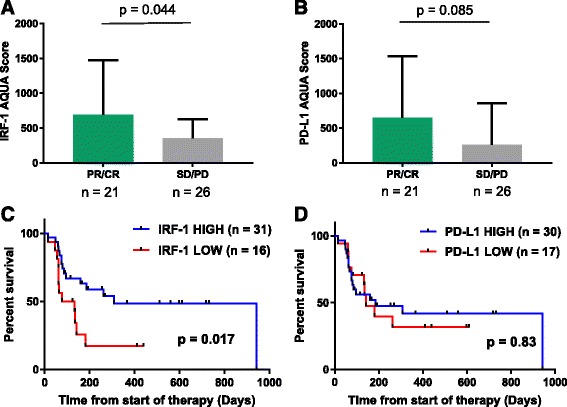
IRF-1 as a predictive marker for anti-PD-1 therapy. **a** IRF-1 expression by best objective radiographic response (ORR) (Mean +/- Std Dev) **b**) PD-L1 by ORR (Mean +/- SD). **c** Progression-free survival from the start of therapy stratified by IRF-1 expression level) **d**) Progression-free survival from the start of therapy stratified by PD-L1 expression level

When classified by best ORR, AQUA scores for nuclear IRF-1 expression was higher in patients with PR/CR than in patients with SD/PD (*p =* 0.044, Fig. 3a). There was a trend toward higher PD-L1 expression in patients with PR/CR (*p =* 0.085, Fig. 3b), though this did not reach statistical significance. We then compared PFS from the start of therapy by IRF-1 expression level. PFS was related to ORR, but there was wide variability in PFS in the PR/CR and SD groups (Additional file 1: Figure S3). Treated cases were then stratified into IRF-1-high and IRF-1-low cohorts using the lowest tertile as the IRF-1-low cohort (AQUA cutpoint = 194). PFS from the start of therapy was significantly higher in the IRF-1-high group than the IRF-1-low group (*p =* 0.017, Fig. 3c). There was a trend toward higher OS in the IRF-1 high group, though this did not reach statistical significance (*p =* 0.060). To determine if there was biologic significance to this cutpoint, we determined the limit of detection for IRF-1 by staining five serum-starved melanoma cell lines for IRF-1 and identifying the lowest AQUA score a FOV with positive nuclear staining. Of five cell lines, only YUSOC had positive IRF-1 staining in the absence of IFNγ; the lowest FOV AQUA score was for YUSOC was 171 (Additional file 1: Figure S4) When the cohort was stratified by this cutpoint, PFS was still higher in the IRF-1-high than the IRF-1-low group (*p =* 0.0386, data not shown). Similarly, cases were stratified into PD-L1-high and PD-L1-low cohorts using a visual cutoff of 120. There was no difference in PFS (*p =* 0.83, Fig. 3d) or OS (*p =* 0.98) between these two cohorts.

**Fig. 4:**
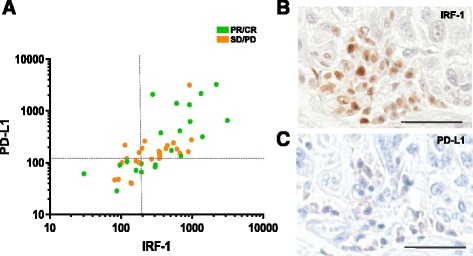
Relationship between PD-L1 and IRF-1 expression. **a** Correlation of IRF-1 with PD-L1 (*p =* 0.002). Dashed lines represent cutoffs between high and low expression cohorts for PD-L1 and IRF-1 **b**), **c**) Serial whole-tissue sections showing chromogenic IRF-1 and PD-L1 IHC staining in a patient in the IRF-1-high, PD-L1-low cohort. Scale bar = 50 uM

PD-L1 expression correlated with IRF-1 expression with a Pearson’s correlation coefficient of 0.52 (Fig. 4a, *p =* 0.0002). However, within the PD-L1-low cohort, four cases were classified as IRF-1-high. An example of one of these cases with high IRF-1 and low PD-L1 is shown in 4B and C. Despite this small sample size, there was a trend toward better PFS in those patients compared to those classified as IRF-1-low, PD-L1-low (*p =* 0.083).

When patients were grouped by therapy (PD-1 inhibitors alone versus the combination with CTLA-4 inhibitors), IRF-1 predicted longer PFS in the combination ipilimumab/nivolumab group (*p =* 0.0051), but this difference did not reach statistical significance in patients treated with single-agent nivolumab or pembrolizumab (*p =* 0.22, Additional file 1: Figure S5). Average IRF-1 AQUA scores were not significantly different for cases treated with combination and single-agent therapy (639 v. 418, *p =* 0.20).

In a Cox proportional hazards model for PFS, IRF-1-low status conferred a hazard ratio of 7.13 (95% Confidence Interval: 1.98–29.55, *p =* 0.0023) when adjusted for age at diagnosis, race, sex, stage group at diagnosis, mutational status, and prior checkpoint blockade.

## Discussion

Biomarkers for predicting response to anti-PD-1 immunotherapy have been identified as a critical unmet need in the treatment of metastatic melanoma [28]. PD-L1 has been shown to be promising in some studies [29] but not others. Here we show pilot data to suggest that capability to express PD-L1 may be more valuable as a predictive marker than PD-L1 itself.

While IRF-1’s role in regulating an inflamed melanoma phenotype has been previously characterized, [21] this is the first report of IRF-1 as a predictive tissue biomarker to anti-PD-1 immunotherapy in melanoma. While the improved clinical responses for tumors with higher IRF-1 expression could reflect these tumors’ ability to express PD-L1, this finding is also consistent with recent studies that have linked IFNγ signaling with response to PD-1[30–33] blockade. Given the role of IRF-1 as a mediator of IFNγ, it is also possible that IRF-1 captures a broader set of tumors suppressing immune effector cells through mechanisms other than PD-L1.

There are a number of limitations to consider for this pilot study. Perhaps the most significant is the small samples size and the fact that the study is a single institutional, retrospective analysis.

Another potential issue is the selection of a cutpoint to distinguish high from low expressers for an assay that results in a continuous data set. Here, we sought to bolster the lowest-tertile cutpoint by also using the limit of detection in unstimulated melanoma cell lines. Using this alternate cutpoint, only three cases were re-classified from the low-IRF-1 to the high-IRF-1 group, and the difference in PFS between IRF-1-high and IRF-1-low patients remained significant. Further development of IRF-1 as a predictive biomarker will require validation of an optimal, reproducibly defined, cutpoint on additional cohorts, as well as inclusion in prospective studies. Also, as the study cohort included patients treated with both single-agent PD-1 and combination PD-1/CTLA-4 blockade, future studies should likely be limited to a more uniform treatment strategy.

In the future development of this assay, stromal expression of IRF-1 should also be considered. While this assay did not include the appropriate markers to accurately quantify IRF-1 in immune cell populations (e.g., CD3), marked differences in stromal cell expression of IRF-1 were noted across cases. Also, it is possible that combination of IRF-1 and PD-L1 or other contributory transcription factors could increase the predictive power of this assay—a multiplex assay including both markers would be most appropriate for testing this hypothesis. In doing so, alternative antibodies for PD-L1 could be considered, as the SP142 clone used in this study has recently been shown to equivalent to other antibodies, including those used in current companion diagnostic tests [34]. However, the SP142 Ventana assay has to be less sensitive than other FDA-approved assays [35]. Here we used the SP142 antibody, but not the Ventana assay.

While the underlying mechanisms remain unclear, there are a number of biological explanations that could explain the association between IRF-1 expression and response to anti-PD-1 immunotherapy. With further validation, it is possible that an IHC-based assay for IRF-1 could be readily transferred to the clinical setting. The concept of a companion diagnostic tested based on capability to express the target of PD-1 axis therapy may address some of the current assays deficiencies related to heterogeneity or other less well defined variables.

## Conclusions

This study is this first report of IRF-1 as a tissue-based biomarker to predict response to anti-PD-1 immunotherapy in melanoma. Compared to PD-L1 status, nuclear IRF-1 staining better predicted objective radiographic response and progression-free survival in a retrospective cohort of 47 melanoma patients. Furthermore, this effect was greatest in patients treated with combination ipilimumab and nivolumab, which is rapidly being adopted as a standard of care. Given the limited utility of PD-L1 as a predictive biomarker in this disease, assays for IRF-1 warrant further investigation in randomized controlled trials to determine if they could serve as clinically useful alternatives to guide treatment decisions.

## Additional file

Additional file 1: Figures S1 through S5. (PPTX 184 kb)

## Abbreviations

BSA: Bovine serum albumin
CR: Complete response
CTLA-4: cytotoxic T-lymphocyte associated protein 4
DAPI: 4’,6 diamidino-2-phenylindole
EtOH: Ethanol
FDA: Food and drug administration
FFPE: Formalin-fixed, paraffin-embedded
FOV: Field of view
IFNγ: Interferon gamma
IHC: Immunohistochemical
IRF-1: Interferon regulatory factor-1
ORR: Objective radiographic response
OS: Overall survival
PBS: Phosphate-buffered saline
PD: Progressive disease
PD-1: Programmed death 1
PD-L1: Programmed death-ligand 1
PFA: Paraformaldehyde
PFS: Progression-free survival
PR: Partial response
RT: Room temperature
SD: Stable disease
TBST: Tris-buffered saline + tween
TMA: Tissue microarray
