# Supplementary material for: Nuclear IRF-1 expression as a mechanism to assess “Capability” to express PD-L1 and response to PD-1 therapy in metastatic melanoma
Source: J Immunother Cancer. 2017 Mar 21;5:25. doi: 10.1186/s40425-017-0229-2 (PMC5359951; doi:10.1186/s40425-017-0229-2)
Supplement: Additional file 1: — Figures S1 through S5. (PPTX 184 kb) [file 40425_2017_229_MOESM1_ESM.pptx]

## Slide 1
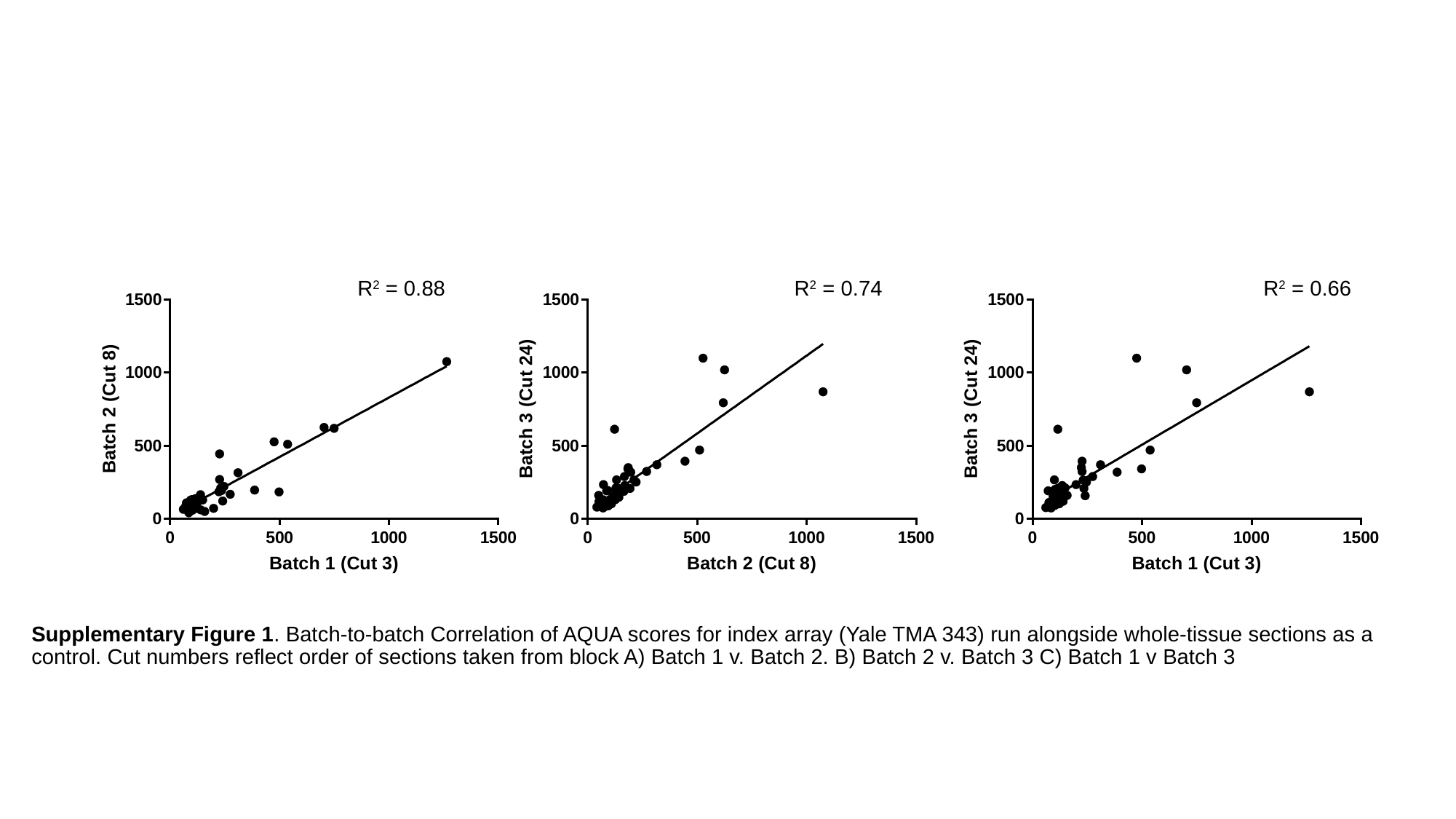

R2 = 0.88
R2 = 0.74
R2 = 0.66
Supplementary Figure 1. Batch-to-batch Correlation of AQUA scores for index array (Yale TMA 343) run alongside whole-tissue sections as a control. Cut numbers reflect order of sections taken from block A) Batch 1 v. Batch 2. B) Batch 2 v. Batch 3 C) Batch 1 v Batch 3

## Slide 2
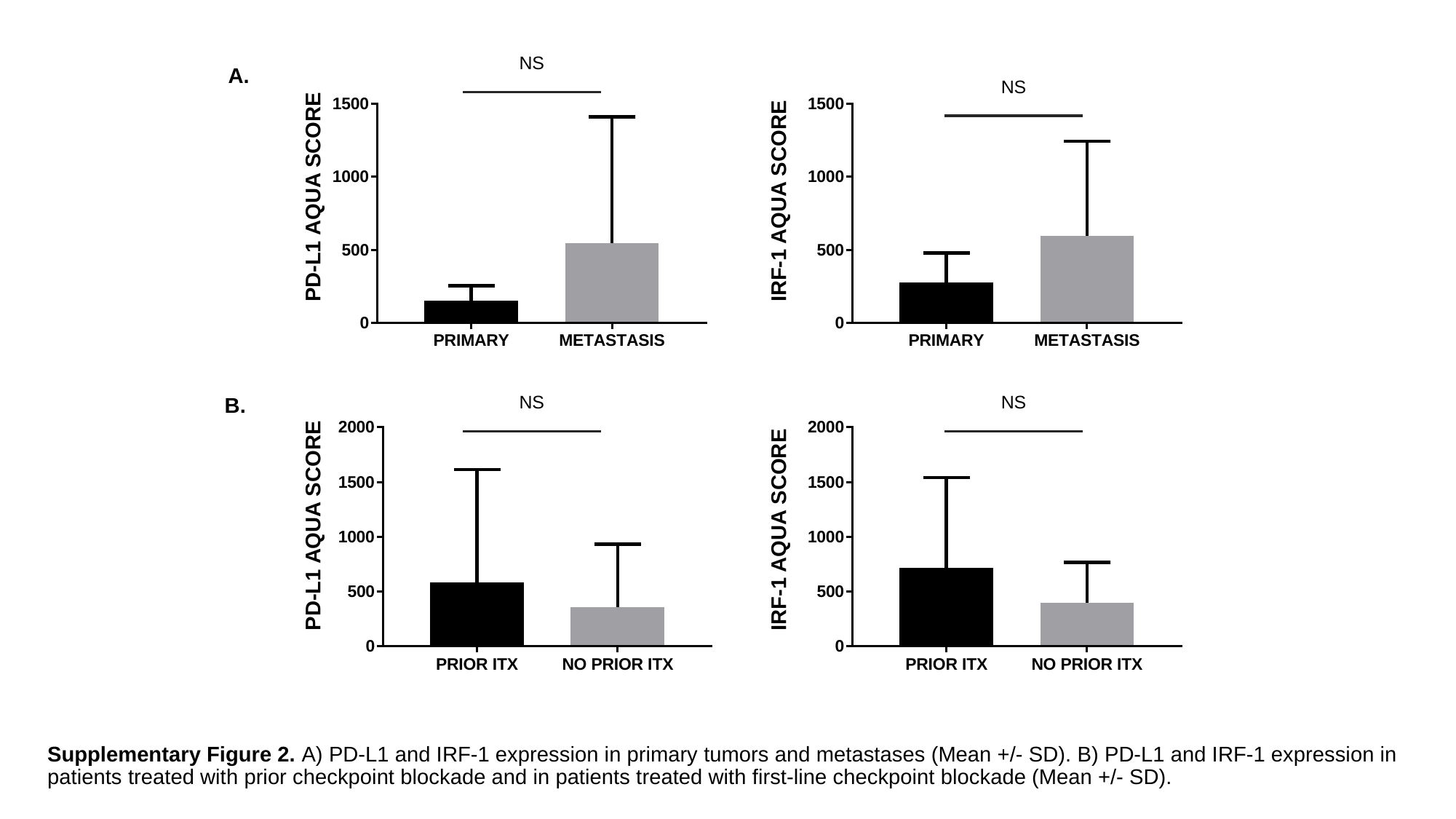

A.
NS
NS
PD-L1 AQUA SCORE
IRF-1 AQUA SCORE
B.
NS
NS
PD-L1 AQUA SCORE
IRF-1 AQUA SCORE
# Supplementary Figure 2. A) PD-L1 and IRF-1 expression in primary tumors and metastases (Mean +/- SD). B) PD-L1 and IRF-1 expression in patients treated with prior checkpoint blockade and in patients treated with first-line checkpoint blockade (Mean +/- SD).

## Slide 3
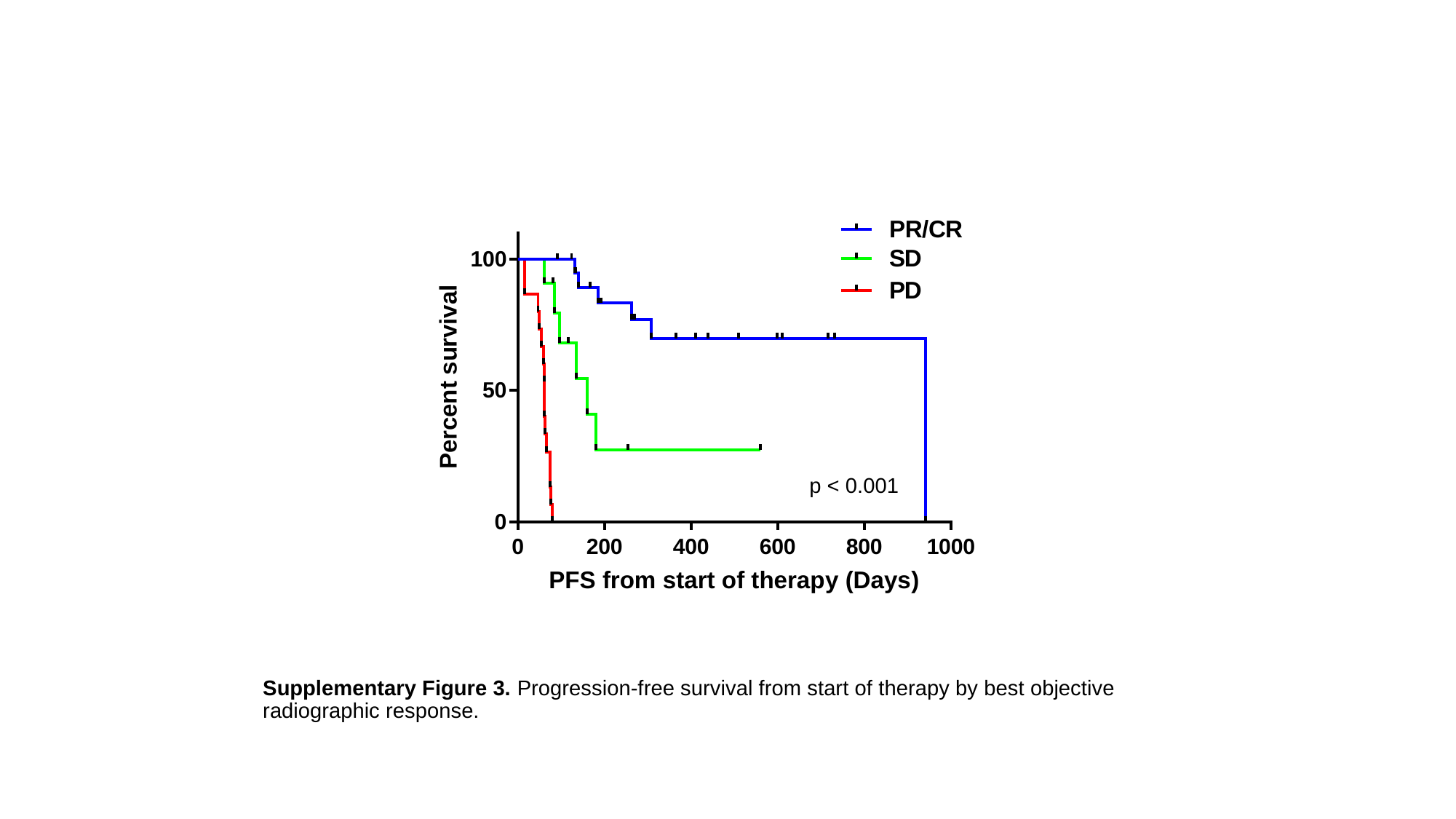

p < 0.001
# Supplementary Figure 3. Progression-free survival from start of therapy by best objective radiographic response.

## Slide 4
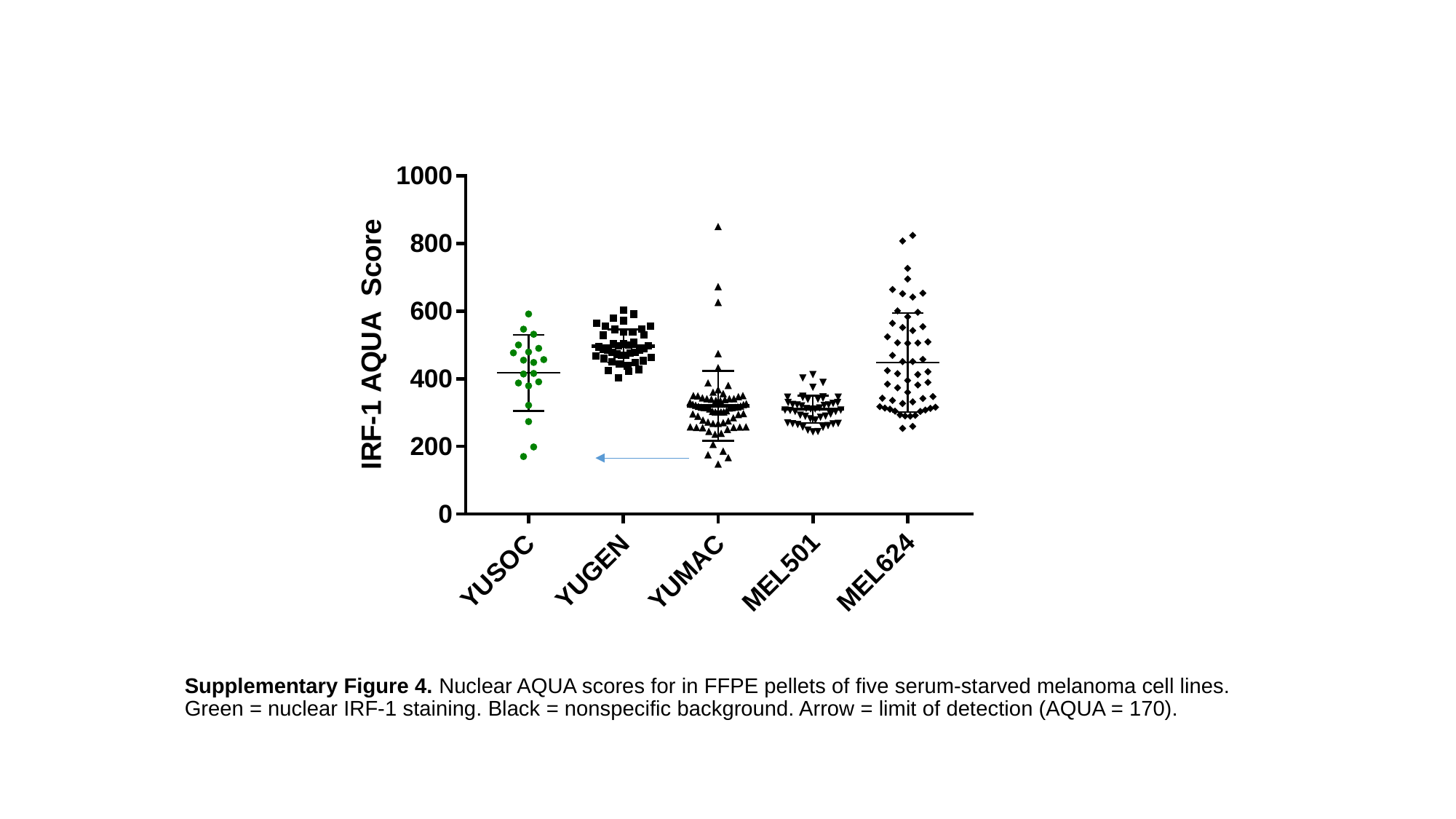

# Supplementary Figure 4. Nuclear AQUA scores for in FFPE pellets of five serum-starved melanoma cell lines. Green = nuclear IRF-1 staining. Black = nonspecific background. Arrow = limit of detection (AQUA = 170).

## Slide 5
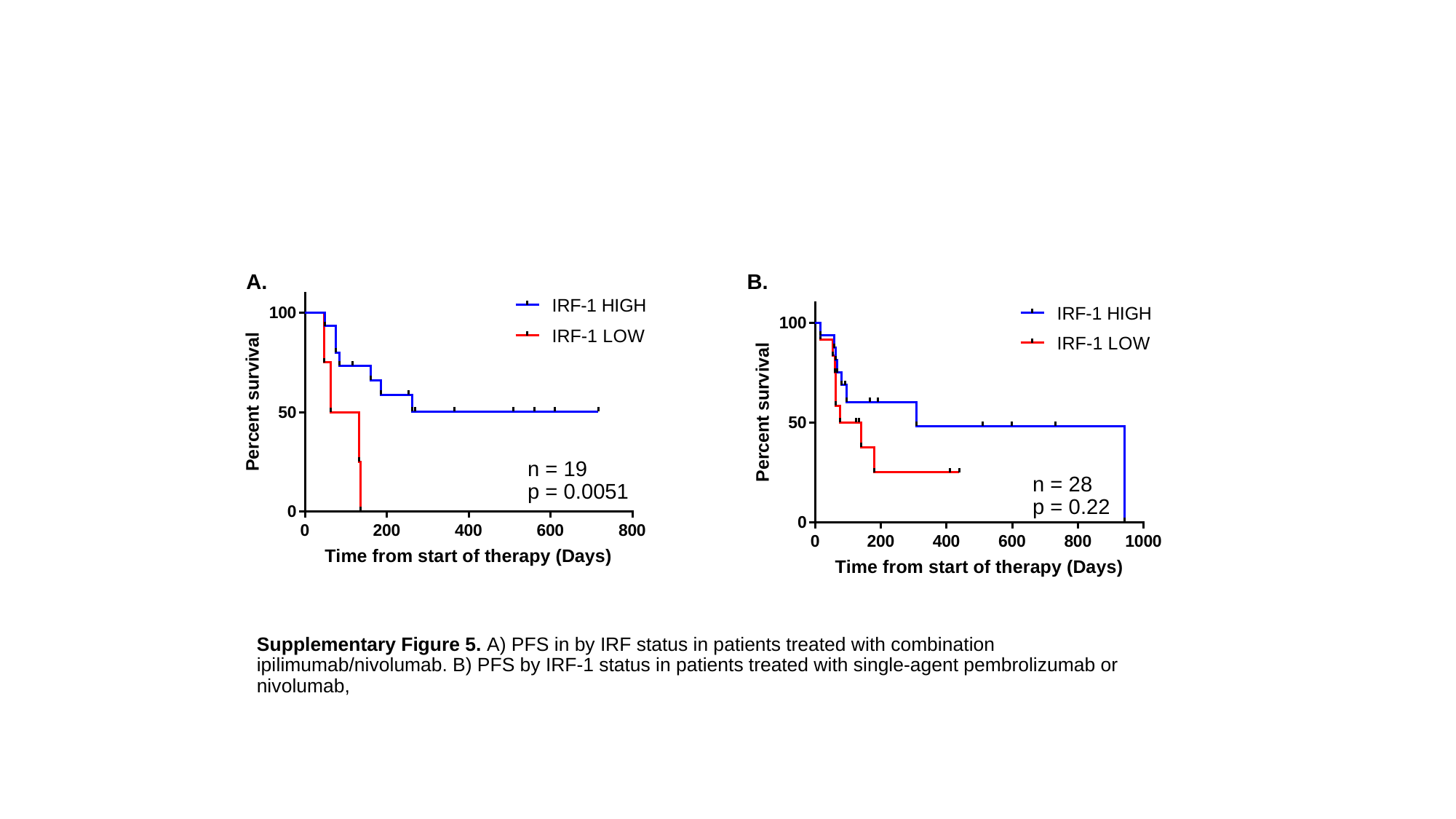

A.
B.
n = 19
p = 0.0051
n = 28
p = 0.22
# Supplementary Figure 5. A) PFS in by IRF status in patients treated with combination ipilimumab/nivolumab. B) PFS by IRF-1 status in patients treated with single-agent pembrolizumab or nivolumab,
